# A novel missense variant in the nuclear localization signal of *POU4F3* causes autosomal dominant non-syndromic hearing loss

**DOI:** 10.1038/s41598-017-08236-y

**Published:** 2017-08-08

**Authors:** Yin-Hung Lin, Yi-Hsin Lin, Ying-Chang Lu, Tien-Chen Liu, Chien-Yu Chen, Chuan-Jen Hsu, Pei-Lung Chen, Chen-Chi Wu

**Affiliations:** 1 Department of Otolaryngology, National Taiwan University Hospital, Taipei, Taiwan; 2 Graduate Institute of Medical Genomics and Proteomics, National Taiwan University College of Medicine, Taipei, Taiwan; 3 Graduate Institute of Molecular Medicine, National Taiwan University College of Medicine, Taipei, Taiwan; 4 Department of Bio-Industrial Mechatronics Engineering, National Taiwan University, Taipei, Taiwan; 5 Department of Otolaryngology, Taichung Tzu-Chi Hospital, Taichung, Taiwan; 6 Department of Medical Genetics, National Taiwan University Hospital, Taipei, Taiwan; 7 Graduate Institute of Clinical Medicine, National Taiwan University College of Medicine, Taipei, Taiwan; 8 Department of Internal Medicine, National Taiwan University Hospital, Taipei, Taiwan

## Abstract

Autosomal dominant non-syndromic hearing loss (ADNSHL) is genetically heterogeneous with more than 35 genes identified to date. Using a massively parallel sequencing panel targeting 159 deafness genes, we identified a novel missense variant of *POU4F3* (c.982A>G, p.Lys328Glu) which co-segregated with the deafness phenotype in a three-generation Taiwanese family with ADNSHL. This variant could be classified as a “pathogenic variant” according to the American College of Medical Genetics and Genomics guidelines. We then performed subcellular localization experiments and confirmed that p.Lys328Glu compromised transportation of POU4F3 from the cytoplasm to the nucleus. *POU3F4* p.Lys328Glu was located within a bipartite nuclear localization signal (NLS), and was the first missense variant in bipartite NLS of POU4F3 validated in functional studies. These findings expanded the mutation spectrum of *POU4F3* and provided insight into the pathogenesis associated with aberrant POU4F3 localization.

## Introduction

Autosomal dominant non-syndromic hearing loss (ADNSHL) is a heterogeneous disease entity with more than 35 genes identified to date (http://hereditaryhearingloss.org/). From an epidemiological perspective, none of these ADNSHL genes is more prevalent than another, making it difficult to perform genetic testing using conventional Sanger sequencing^1^. Recently, massively parallel sequencing (MPS), also known as targeted next-generation sequencing (NGS), has been proven to be a powerful tool in addressing genetically heterogeneous hereditary hearing impairment^2^. By using MPS-based panels, genetic causes could be determined in >50% of cases with ADNSHL^3^.

*POU4F3* (MIM #602460) is one of the earliest deafness genes identified to cause ADNSHL DFNA15. In 1998, Vahava et al. detected an 8-base pair deletion in *POU4F3* in a five-generation Israeli Jewish family^4^. To date, only 12 *POU4F3* causative variants have been reported in the literature, that is, six missense variants^5–10^, five frameshift deletions^4, 11–14^, and a large deletion encompassing the entire gene^15, 16^. *POU4F3* is located on 5q31 and encodes a protein of 338 amino acids, which functions as a transcription factor with two DNA-binding domains: the POU-specific domain and the POU homeodomain^17^. Two nuclear localization signals (NLSs) crucial for active protein transport into the nucleus are located within the POU homeodomain. The first is a monopartite NLS located at amino acids 274 to 278, and the second is a bipartite NLS located at amino acids 314 to 331^18^. According to previous reports, mutations in *POU4F3* might lead to late-onset bilateral progressive hearing loss with down-sloping audiometric configurations^8^.

## Results

### Clinical features

**Figure 1:**
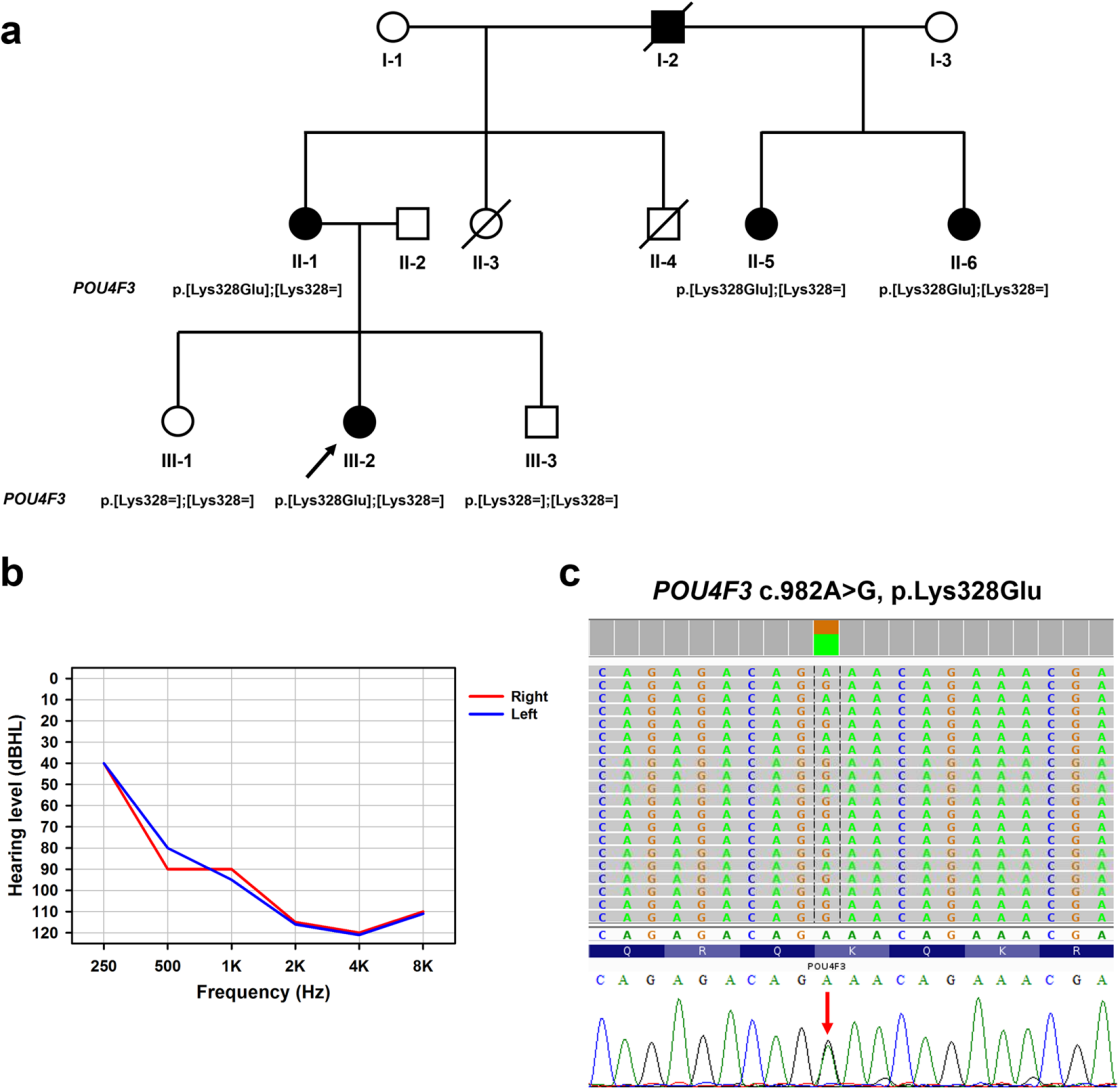
(**a**) The pedigree and segregation pattern of the family, which harbored *POU4F3* p.Lys328Glu. (**b**) The audiogram of both ears in the proband of the family revealed profound hearing loss with down-sloping shape. Hearing levels of the right and the left ear are marked using red and blue lines, respectively. (**c**) MPS-based panel identified a *POU4F3* c.982A>G, p.Lys328Glu variant in the proband and this variant was validated by Sanger sequencing.

The proband of the family was a 45-year-old woman who had bilateral progressive hearing impairment since around 30 years of age. Audiogram of the better ear of the proband showed the following hearing acuity: 40 dB at 250 Hz, 80 dB at 500 Hz, 90 dB at 1000 Hz, 115 dB at 2000 Hz, 120 dB at 4000 Hz, and 110 dB at 8000 Hz. Audiometry showed a down-sloping configuration (Fig. 1b). Her grandfather, her mother, and her mother’s two half-sisters (with the same father) also presented with late onset progressive hearing impairment (Fig. 1a).

### Identification of the causative variant

Using MPS-based panel, the average depth of coverage reached 134 folds, with 99.7% of sequences having coverage greater than one fold, 90.2% greater than 30 folds, and 63.8% greater than 100 folds. There were 333 variants located within exons or splice sites of the targeted 159 genes, nearly half of them were synonymous (166 out of 333). After excluding synonymous variants, there were nine variants, including seven nonsynonymous variants and two non-frameshift deletions, with allele frequencies less than 0.5% in the NHLBI-ESP 6500 exome project, 1000 Genomes project, and the East Asian population of the ExAC project (Supplementary Table S1). Prediction of pathogenicity using seven algorithms concluded that *POU4F3* p.Lys328Glu (c.982A>G) was the only candidate variant that matched the inheritance pattern.

The c.982A>G is in exon 2 of *POU4F3* (NM_002700.2). We performed Sanger sequencing (Fig. 1c) and found *POU4F3* c.982A>G co-segregated with the deafness phenotype in the family (Fig. 1a). *POU4F3* c.982A>G was absent in the NHLBI-ESP 6500 exome project, 1000 Genomes project, ExAC project, 100 normal-hearing Han Chinese controls, and Taiwan Biobank database. All seven of the algorithms reported deleterious effects of the *POU4F3* p.Lys328Glu.

### Effect of p.Lys328Glu on subcellular localization of POU4F3

**Figure 2:**
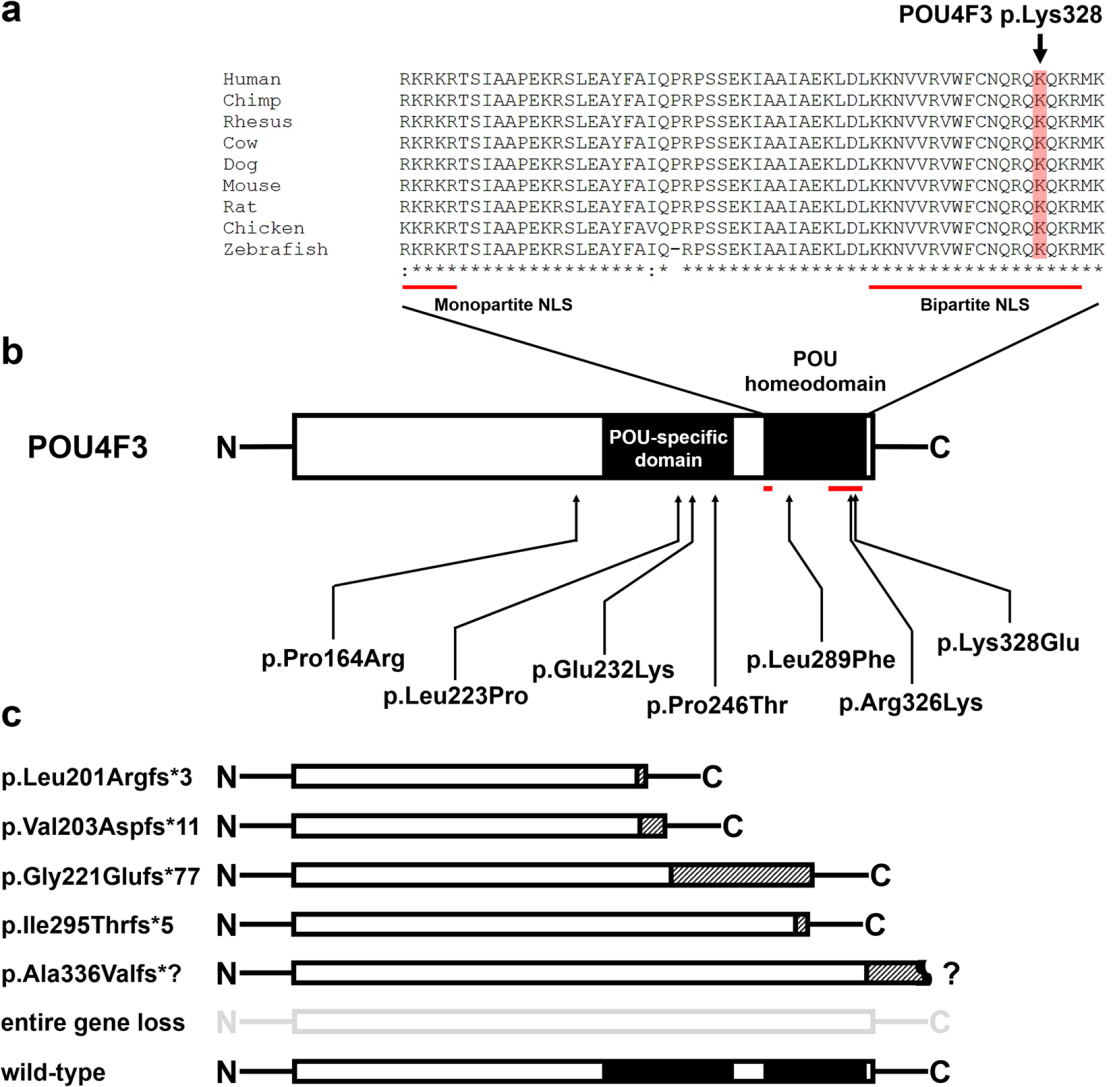
(**a**) The POU4F3 p.Lys328 indicated in red shading was located within the bipartite nuclear localization signal (NLS) in the POU homeodomain. The bipartite NLS was conserved across nine species. (**b**) The location of six reported *POU4F3* missense variants and p.Lsy328Glu. POU-specific domain and POU homeodomain are indicated by black boxes. Two NLSs were indicated by red lines. (**c**) The predicted protein products of five reported *POU4F3* frameshift variants. Boxes with slash indicate the new amino acid sequences after the frameshift.

**Figure 3:**
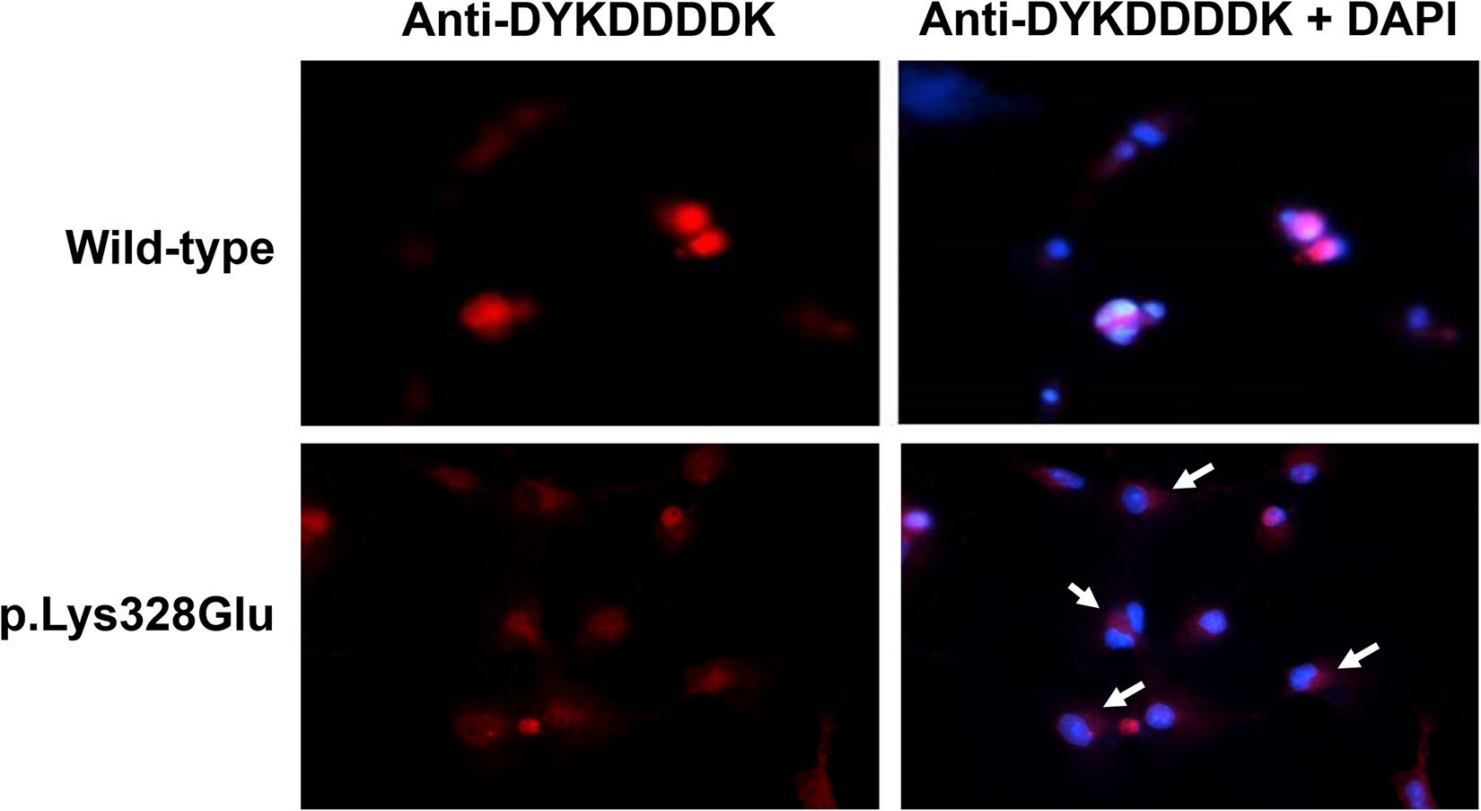
The *POU4F3* c.982A>G mutation altered the subcellular localization of transcription factor POU4F3. Nuclei stained with DAPI (blue) and POU4F3 detected by Anti-DYKDDDDK-Tag antibody (red) were visualized by confocal microscopy. The white arrows indicate POU4F3 outside the nuclei.

The p.Lys328Glu is located within the bipartite NLS of the conserved POU homeodomain (Fig. 2a), suggesting the variant might affect the subcellular localization of POU4F3^18^. To confirm this, we expressed the wild-type or mutant POU4F3 in COS-7 cells. As shown in Fig. 3, whereas the wild-type POU4F3 was located almost exclusively in the nucleus, a portion of the mutant POU4F3 was retained in the cytoplasm, indicating the localization ability of the mutant protein was compromised. Quantification of the transfected cells revealed that the percentage of cells with mutant POU4F3 in the cytoplasm (88%) was much higher than that of cells with the wild-type protein in the cytoplasm (19%).

### Pathogenicity of *POU4F3* p.Lys328Glu

When classified according to the American College of Medical Genetics and Genomics (ACMG) guidelines^19^, *POU4F3* p.Lys328Glu was predicted as deleterious by multiple *in silico* algorithms, meeting the PP3 category; it co-segregated with deafness in the family, fulfilling the PP1 category; it was absent from controls in multiple population databases and located in a critical functional domain, thus fitting the PM2 and PM1 categories, respectively; and most importantly, our *in vitro* functional studies demonstrated that this variant compromised protein function, fulfilling the PS3 category. In summary, *POU4F3* p.Lys328Glu met five criteria, namely, PS3, PM1, PM2, PP1, and PP3, and could be classified as a “pathogenic variant” according to the ACMG guidelines.

### Prevalence of *POU4F3* variants in hearing-impaired families

To determine whether *POU4F3* variants cause deafness in other Taiwanese families, we sequenced both exons of *POU4F3* in 13 additional unrelated families with ADNSHL by either Sanger sequencing or the MPS panel. None of these families harbored causative variants in *POU4F3*. Similarly, we did not identify any causative variants of *POU4F3* in additional 210 hearing-impaired families, which have been subjected to our MPS-based panel for genetic testing, that is, 30 autosomal recessive, four X-linked, and 176 sporadic families. In another paper describing the MPS results of 1119 patients with hearing loss, *POU4F3* was not included in the 49 genes in which pathogenic variants were detected^3^. In other words, variants in *POU4F3* are a rare cause of deafness from an epidemiological perspective.

## Discussion

In this study, we identified a novel missense variant *POU4F3* p.Lys328Glu causing ADNSHL in a Taiwanese family. Functional experiments was performed to evaluate the pathogenesis of the mutant protein, and the mislocalization of the protein was detected.

Among the 13 *POU4F3* causative variants documented in the literature, *POU4F3* p.Lys328Glu was the second missense mutation identified within the bipartite NLS (amino acids 314–331) of the POU homeodomain (Fig. 2b,c). Prior to this study, Kim *et al*. reported p.Arg326Lys in a Korean family with late-onset ADNSHL, but no functional studies were performed to examine the effect of p.Arg326Lys on the subcellular localization of POU4F3^8^. Previous *in vitro* experiments have demonstrated defected nuclear localization of POU4F3 if variants result in a truncated protein lacking the bipartite NLS alone^18^ or both mono- and bipartite NLSs^11^. Our findings further add that missense variants involving key amino acid residues within NLSs might also affect POU4F3 protein trafficking.

Monopartite NLSs contain one cluster of basic amino acids, whereas bipartite NLSs are composed of two clusters of basic amino acids separated by a linker^20^. POU4F3 contains both mono- and bipartite NLSs: the sequence of the monopartite NLS is RKRKR, and the sequence of the bipartite NLS is KKNVVRVWFCNLQRQKQKR^18^. Weiss *et al*. demonstrated that the first two (KK) and last two (KR) amino acids of bipartite NLS were crucial for the nuclear localization of POU4F3. The causative missense variant p.Lys328Glu identified in this study corresponded to the fourth last amino acid of the bipartite NLS. It is conceivable that the replacement of a basic lysine with an acidic glutamate significantly changes the molecular characteristics of the bipartite NLS, indicating that correct alignment of the basic amino acids clusters within NLSs is essential for protein localization.

In addition to mislocalization, mutations in *POU4F3* might also affect protein stability. Weiss *et al*. revealed that POU4F3 is a very short-lived protein and the half-life of the mutant protein (p.Ile295Thrfs*5) is longer than that of the wild-type protein^18^. Another study by Collin *et al*. examined the stability of two mutant POU4F3 proteins with missense variants, p.L223P and p.L289F. The stability of these mutant proteins was not different from that of the wild-type protein^5^. Recent studies have demonstrated a novel function of NLS in regulating protein stability through ubiquitin/proteasome system^21, 22^. Deletion of NLS containing lysine ubiquitination sites could decrease protein degradation^21^. The p.Ile295Thrfs*5 mutation disrupted the bipartite NLS of POU4F3, whereas p.L223P and p.L289F did not. This may explain the extended half-life of POU4F3 with the p.Ile295Thrfs*5 mutation. The p.Lys328Glu mutation identified in this study may also affect POU4F3 degradation, because of the substitution of a lysine residue for a glutamic acid residue within the NLS. Further studies are needed to elucidate the effects of different mutations on the stability of POU4F3.

The pathogenetic mechanisms underlying hearing impairment of patients with *POU3F4* variants remain unclear. The molecular mechanisms of dominant inheritance includes haploinsufficiency, gain of function and dominant-negative effect^23^. As a dominant-negative effect has been ruled out, haploinsufficiency is the most likely mechanism so far^5, 18^. Although heterozygous *Pou4f3* knockout mice exhibited normal hearing comparable to wild-type mice^24^, the mechanism of haploinsufficiency has been supported by two different studies. Deletion of the entire *POU4F3* has been reported in a Brazilian family with ADNSHL^15^. A Japanese study also identified a *POU4F3* frameshift variant (c.1007del), which would produce a transcript without in-frame stop codon (p. Ala336Valfs*? in Fig. 2c)^12^, and presumably the nonstop mRNAs might be degraded through non-stop decay^25^. In other words, both variants caused the loss of one copy of *POU4F3*, indicating the mechanism of haploinsufficiency. The subcellular protein mislocalization shown in this study and others^5, 11, 18^ also supports the mechanism of haploinsufficiency.

In conclusion, by using an MPS-based genetic testing panel targeting 159 known deafness genes, we identified a novel *POU4F3* pathogenic variant, p.Lys328Glu, in a Taiwanese family with ADNSHL. *POU4F3* p.Lys328Glu interrupted the bipartite NLS and prevented the transportation of POU4F3 from the cytoplasm to the nucleus. These findings expanded the mutation spectrum of the rare deafness gene *POU4F3*, and provided insights into the pathogenetic mechanisms associated with aberrant POU4F3 localization.

## Methods

### Subjects and clinical evaluation

A three-generation deafness family with five affected members was recruited in the study (Fig. 1a). Comprehensive family history; previous medical records; and results of physical, neurological, audiological examinations were obtained and analyzed. Audiological results were characterized with respect to two parameters, namely, hearing levels and audiogram shapes^26^. Hearing level of the better ear, which was calculated using a 4-tone average (0.5, 1, 2, and 4 kHz), was labeled as mild (20–40 dBHL), moderate (41–70 dBHL), severe (71–95 dBHL), or profound ( > 95 dBHL) hearing loss (GENDEAF: http://audiology.unife.it/www.gendeaf.org/index.html). Informed consent was obtained from all participants and all the procedures used in the study were approved by the Research Ethics Committee of the National Taiwan University Hospital. All methods were performed in accordance with the relevant guidelines and regulations.

### Targeted MPS-based deafness panel

Genomic DNA of the proband was extracted from peripheral blood and subjected to an MPS-based deafness panel targeting 159 known deafness genes. The sequencing methods and analysis pipelines have been previously reported^27, 28^. In brief, genomic DNA was fragmented into 800 bps. After sample preparation, DNA fragments were enriched using custom probes designed to capture 1,299,144 base pairs with target regions encompassing 3,647 coding and non-coding exons of 159 deafness genes. Paired-end sequencing was performed by the Illumina Miseq platform (Illumina Inc., San Diego, CA, USA), which produced 300 bps reads.

### Data analysis and filtering

We used BWA-MEM, Picard, GATK, and ANNOVAR to perform reads mapping, converting, sorting, variants calling, and annotation. Data filtering was conducted by an in-house perl script with the following steps: selection of variants located in the targeted 159 genes; filtering out of variants with allele frequencies more than 0.5% in the NHLBI-ESP 6500 exome project (http://evs.gs.washington.edu/EVS/), 1000 Genomes project (http://www.1000genomes.org/), Exome Aggregation Consortium (ExAC) projects (http://exac.broadinstitute.org/), 100 normal-hearing Han Chinese subjects, and Taiwan Biobank database (https://taiwanview.twbiobank.org.tw/); prediction of the pathogenicity of retained missense variants by seven algorithms including PolyPhen-2, SIFT, LRT, MutationTaster, MutationAssessor, FATHMM, and MetaLR; and confirmation that candidate variants are located in conserved regions of nine species and co-segregate with the deafness phenotype.

### Sanger sequencing

Genomic DNA was extracted from peripheral blood or saliva samples of four affected members and two unaffected members. Sanger sequencing was performed to validate the variants identified by the MPS panel and to examine the co-segregation of variants with deafness among the family members. Allele frequencies of variants segregating with the phenotype were also verified in a panel of 100 normal-hearing Han Chinese subjects.

### Cell transfection and immunocytochemistry

COS-7 cells were transiently transfected by a lentivirus with constructs encoding either the wild-type or the mutant fusion protein pWPT-POU4F3-6XHis-Flag. Transfected COS-7 cells were fixed in 4% paraformaldehyde, permeabilized in 0.5% Triton X-100, and blocked in 10% goat serum. After incubation with DYKDDDDK Tag Antibody (Alexa Fluor 594 Conjugate, Thermo Fisher Scientific, Waltham, MA, USA), the samples were examined with a laser scanning confocal microscope (Zeiss LSM 510, Carl Zeiss, Germany). For quantitative analysis, 80-100 cells with mutant or wild-type POU4F3 mislocalization were counted, and the percentage of cells expressing POU4F3 in the cytoplasm was calculated.

## Electronic supplementary material

Supplementary Information
