# Supplementary material for: A novel missense variant in the nuclear localization signal of POU4F3 causes autosomal dominant non-syndromic hearing loss
Source: Sci Rep. 2017 Aug 8;7:7551. doi: 10.1038/s41598-017-08236-y (PMC5548901; doi:10.1038/s41598-017-08236-y)
Supplement: Supplementary file 1 — Supplementary Information [file 41598_2017_8236_MOESM1_ESM.doc]

**A novel missense variant in the nuclear localization signal of *POU4F3* causes autosomal dominant non-syndromic hearing loss**

Yin-Hung Lin^1,2^, Yi-Hsin Lin^1,3^, Ying-Chang Lu^1^, Tien-Chen Liu^1^, Chien-Yu Chen^4^, Chuan-Jen Hsu^1,5^*, Pei-Lung Chen^2,3,6,7,8^*, Chen-Chi Wu^1,6^*

1. Department of Otolaryngology, National Taiwan University Hospital, Taipei, Taiwan
2. Graduate Institute of Medical Genomics and Proteomics, National Taiwan University College of Medicine, Taipei, Taiwan
3. Graduate Institute of Molecular Medicine, National Taiwan University College of Medicine, Taipei, Taiwan
4. Department of Bio-Industrial Mechatronics Engineering, National Taiwan University, Taipei, Taiwan
5. Department of Otolaryngology, Taichung Tzu-Chi Hospital, Taichung, Taiwan
6. Department of Medical Genetics, National Taiwan University Hospital, Taipei, Taiwan
7. Graduate Institute of Clinical Medicine, National Taiwan University College of Medicine, Taipei, Taiwan
8. Department of Internal Medicine, National Taiwan University Hospital, Taipei, Taiwan

* Address correspondence to: Department of Otolaryngology, Taichung Tzu Chi Hospital, Buddhist Tzu Chi Medical Foundation, 66 Fung-Hing Road Sec. 1, Tanzi District, Taichung, 427, Taiwan (C-J. H.), Graduate Institute of Medical Genomics and Proteomics, National Taiwan University College of Medicine, 1, Sec.1, Jen-Ai Road, Taipei, 100, Taiwan (P-L. C.) & Department of Otolaryngology, National Taiwan University Hospital, 7 Chung-Shan S. Rd., Taipei, 100, Taiwan (C-C. W.).

Fax: 886-2-23410905 (C-J. H. & C-C. W.) & 886-2-33936523 (P-L. C.)

Tel.: +886-2-23123456 ext 65220 (C-J. H.), 71942 (P-L. C.) & 63524 (C-C. W.)

E-mail address: cjhsu@ntu.edu.tw, paylong@ntu.edu.tw & chenchiwu@ntuh.gov.tw

**Table S1. Protein-altering variants with <0.5% ExAC East Asian frequency.**

| **Gene** | **Inheritance** | **Affected transcripts (RefSeq)** | **Affected proteins** | **Genotype** | **Allele frequencies** | | | **Pathogenicity prediction** | | | | | | |
| --- | --- | --- | --- | --- | --- | --- | --- | --- | --- | --- | --- | --- | --- | --- |
|  |  |  |  |  | **ESP 6500** | **1000 Genomes** | **ExAC East Asian** | **SIFT^a^** | **PolyPhen-2^b^ (HumVar)** | **LRT** | **MutationTaster^c^** | **MutationAssessor^d^** | **FATHMM** | **MetaLR** |
| *USH2A* | AR | c.7000A>G (NM_206933.2) | p.Asn2334Asp | Het | NA | 0.0002 | 0.0013 | 0.701 (T) | 0.104 (B) | Neutral | Polymorphism | Low | Tolerated | Tolerated |
| *ALMS1* | AR | c.72_77del (NM_015120.4) | p.Glu28_Glu29del | Het | NA | NA | NA | NA | NA | NA | NA | NA | NA | NA |
| *DSPP* | AD | c.2661A>C (NM_014208.3) | p.Glu887Asp | Het | NA | 0.000599 | 0 | NA | 0 (B) | NA | Polymorphism | Neutral | **Damaging** | Tolerated |
| *POU4F3* | AD | c.982A>G (NM_002700.2) | p.Lys328Glu | Het | NA | NA | NA | **0 (D)** | **1 (D)** | **Deleterious** | **Disease causing** | **High** | **Damaging** | **Deleterious** |
| *SERPINB6* | AR | c.469G>C (NM_001271823.1) | p.Val157Leu | Het | NA | NA | 0.0001 | **0.001 (D)** | 0.765 (P) | **Deleterious** | **Disease causing** | **High** | **Damaging** | **Deleterious** |
| *FGF3* | AR | c.404G>A (NM_005247.2) | p.Arg135Gln | Het | NA | NA | 0.0009 | 0.093 (T) | 0.142  (B) | Neutral | **Disease causing** | Low | Tolerated | Tolerated |
| *STRC* | AR | c.52_54del (NM_153700.2) | p.Leu18del | Het | NA | NA | NA | NA | NA | NA | NA | NA | NA | NA |
| *OTOA* | AR | c.35T>G (NM_144672.3) | p.Leu12Arg | Het | NA | NA | 0.0001 | **0 (D)** | **0.999 (D)** | **Deleterious** | **Disease causing** | **Medium** | Tolerated | Tolerated |
| *TRIOBP* | AR | c.4097C>T (NM_001039141.2) | p.Thr1366Ile | Het | NA | NA | 0.0002 | 0.079 (T) | 0.004  (B) | NA | Polymorphism | Neutral | Tolerated | Tolerated |

AD, autosomal dominant; AR, autosomal recessive; Het, heterozygosity; NA, not available.

^a^ D and T of SIFT stand for “Deleterious” and “Tolerated”, respectively.

^b^ D, P and B of PolyPhen-2 stand for “Probably damaging”, ”Possibly damaging”, and “Benign”, respectively.

^c^ Disease causing and Polymorphism in MutationTaster mean “probably deleterious” and “probably harmless”, respectively.

^d^ MutationAssessor predicts the functional impact of missense mutations with four levels (High, Medium, Low, and Neutral).

Predictions as pathogenic variants are marked in bold.
